# Supercritical Carbon Dioxide-Processed Acellular Dermal Matrix Patch for Enhanced Wound Healing

**DOI:** 10.3390/ijms26125715

**Published:** 2025-06-14

**Authors:** Xinrui Zhang, Linh Thi Thuy Le, Yongxun Jin, Caijun Jin, Nguyen Ngan Giang, Thuy-Tien Thi Trinh, Yong Hyun Lee, Yong Woo Shin, Jin Woo Bae, Pham Ngoc Chien, Chan Yeong Heo

**Affiliations:** 1 Department of Plastic and Reconstructive Surgery, College of Medicine, Seoul National University, Seoul 03080, Republic of Korea; 2 Department of Plastic and Reconstructive Surgery, Seoul National University Bundang Hospital, Seongnam 13620, Republic of Korea; 3 Department of Biomedical Science, College of Medicine, Seoul National University, Seoul 03080, Republic of Korea; 4 Department of Medical Technology, Haiphong University of Medicine and Pharmacy, Haiphong 180000, Vietnam; 5 Department of Medical Device Development, College of Medicine, Seoul National University, Seoul 03080, Republic of Korea; 6 Korean Institute of Nonclinical Study (KINS), Seongnam 13605, Republic of Korea; 7 DOF Inc., Hwaseong 18468, Republic of Korea

**Keywords:** supercritical carbon dioxide, acellular dermal matrix, patch, wound healing

## Abstract

Wound healing remains a significant clinical challenge worldwide, and effective management strategies are essential for improving outcomes. This study evaluated SCderm Matrix, a novel acellular dermal matrix (ADM) patch developed using supercritical carbon dioxide (sCO_2_) processing of human skin tissue. This innovative processing method preserves structural integrity while enhancing biocompatibility, resulting in a patch characterized by porous architecture, uniform thickness, excellent tensile strength, and optical transparency. In vivo wound healing experiments using full-thickness skin wounds in Sprague–Dawley rats demonstrated the patch’s superior performance. Treatment with the sCO_2_ ADM patch accelerated wound closure, reduced inflammation, and enhanced granulation tissue formation compared to both untreated controls and two commercially available ADM products. Histological analysis revealed improved re-epithelialization and collagen deposition, while molecular and immunohistochemical assessments showed decreased reactive oxygen species (ROS) and pro-inflammatory cytokines. Simultaneously, the treatment upregulated key proliferation and remodeling markers including alpha smooth muscle actin (α-SMA), vimentin, and transforming growth factor beta 1 (TGF-β1). These findings demonstrate that the SCderm Matrix promotes wound healing through multiple mechanisms: modulating inflammatory responses, enhancing antioxidant defenses, and supporting tissue regeneration. The results suggest this biomaterial has significant potential as an effective and versatile solution for clinical wound care applications.

## 1. Introduction

The skin, with the dermis and epidermis serving as the body’s primary protective barrier, plays a critical role in multiple physiological processes, including immunological defense, environmental sensing, and thermoregulation [1,2,3,4,5,6,7,8,9]. Due to its continuous exposure and dynamic interface with external environments, cutaneous tissue is inherently vulnerable to disruption, manifesting as wound formations [10,11,12,13,14]. Wound healing is a complex, multifaceted process involving a series of orchestrated phases—hemostasis, inflammation, proliferation, and remodeling—designed to restore the integrity and functionality of injured tissue [15,16,17,18,19,20]. Despite the skin’s intrinsic regenerative capacities and wound repair mechanisms, certain conditions, such as diabetes, vascular insufficiency, and aging, often lead to chronic wounds characterized by prolonged inflammation, oxidative stress, inadequate vascularization, and fibrosis and complicates the repair mechanisms and increasing the risk of complications [21,22,23,24,25]. This highlights the need for advanced therapeutic strategies that can modulate the microenvironment and promote angiogenesis.

Research on using extracellular matrix (ECM) scaffolds to facilitate wound healing has significantly advanced since the late 20th century, with substantial progress made in the early 21st century [26,27,28,29,30,31,32,33,34]. Compositionally, ECM comprises a sophisticated network of biological molecules such as fibrin, fibronectin, various collagen types, and hyaluronic acid, which collectively facilitate dermal regeneration and provide an optimal structural framework for tissue remodeling. However, native ECM compositions present significant immunological challenges because the multifaceted cellular diversity inherent in these matrices can potentially trigger inflammatory responses and immunologic reactions [35,36,37,38]. In order to mitigate these risks, researchers strategically eliminate immunogenic cellular elements through decellularization process to create biologically compatible scaffolds, namely decellularized extracellular matrices (dECMs) [39,40,41,42,43].

Acellular dermal matrices (ADMs), as a form of dECMs, have garnered attention for their ability to enhance wound healing through their biocompatibility, immunomodulatory properties, and capacity to support cellular integration and angiogenesis [44,45,46,47,48]. ADMs are primarily composed of collagen, elastin, and preserved growth factors, offering low immunogenicity and high biocompatibility. They provide a structural scaffold for cell migration, angiogenesis, and tissue remodeling [49,50,51,52]. ADM preparation techniques, such as decellularization, must balance the removal of immunogenic components with the preservation of native ECM architecture [40,53,54]. Traditionally, the decellularization of animal tissues involves a combination of physical, chemical, and biological methods, such as using acid–base solvents, detergents, enzymatic treatments, or repeated freeze–thaw cycles. However, in these decellularization methods, preventing the loss and degradation of extracellular matrix components due to chemical reagents is a challenge. Some key substances in the extracellular matrix, such as glycoproteins and growth factors, are crucial for tissue regeneration, but they may be denatured or removed due to the surfactant action of detergents [55,56]. Carbon dioxide molecules in the supercritical state can penetrate tissues, using their dissolving capability to dissolve certain intracellular components, thereby removing cellular contents. Additionally, supercritical carbon dioxide (sCO_2_) can increase cell membrane fluidity, accelerate cell membrane rupture, release cellular contents, and further promote the decellularization process [57,58]. Since it does not involve chemical reagent usage and can be operated at lower temperatures and pressures, decellularization based on sCO_2_ can largely preserve bioactive compounds and the structure of extracellular matrices while removing immunogenic substances, demonstrating clear advantages over traditional decellularization methods [59,60]. Moreover, before using the material, the most critical step is to ensure thorough sterilization to prevent infection and immune reactions, thus enhancing safety. SCO_2_ can avoid structural damage to or chemical degradation of the extracellular matrix. Due to its chemical inertness, high dissolving capacity, low viscosity, and high diffusion coefficient, sCO_2_ can uniformly and rapidly inactivate drug-resistant bacterial spores, viruses, and fungi without introducing toxic by-products [58,61,62,63,64,65,66,67,68]. In addition, the acellular dermal matrix powder was mixed with sterile distilled water to create a suspension, which was then spread and dried to form a patch. This processing method significantly enhanced tensile strength, making the patch more suitable for clinical applications.

We employed sCO_2_ as a method for decellularization to prepare human-derived ADMs. ADMs were freeze-ground into microparticles, which were then combined with sterile distilled water. Through a high-pressure dispersion process followed by vacuum drying, we successfully fabricated ADM patches and then evaluated their properties and safety through in vitro testing. Subsequently, we used Sprague–Dawley rats as an animal model to assess the efficacy of these matrices in skin wound healing. Our findings demonstrated an effective reduction in oxidative stress and inhibition of pro-inflammatory cytokine expression. This approach successfully improved the wound microenvironment by reducing inflammation, ultimately promoting healing.

## 2. Results

### 2.1. Characterization of sCO_2_ ADM Patch

**Figure 1:**
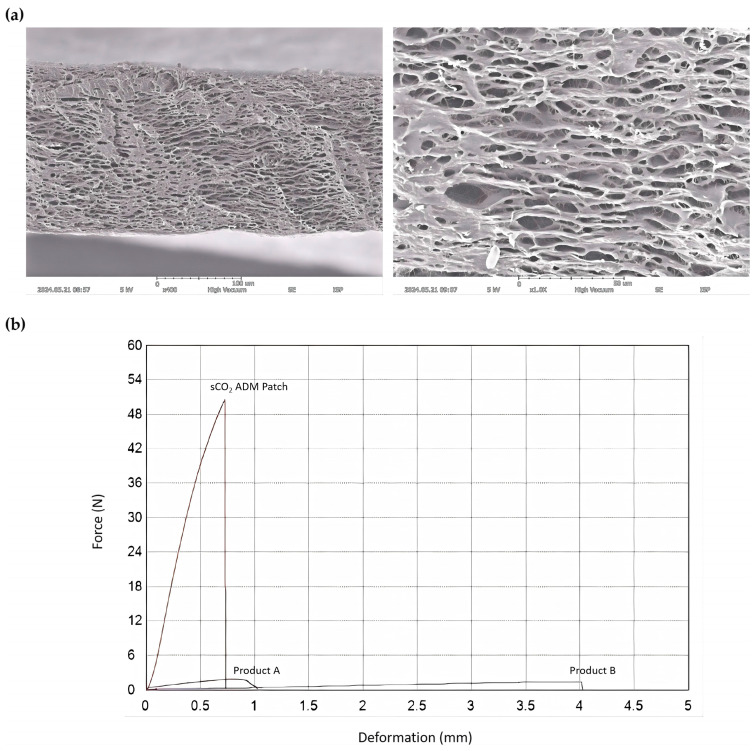
(**a**) Observation of sCO_2_ ADM patch’s structural properties using a scanning electron microscope under 400× and 1000× magnification. (**b**) Tensile strength of different ADM patches.

**Figure 2:**
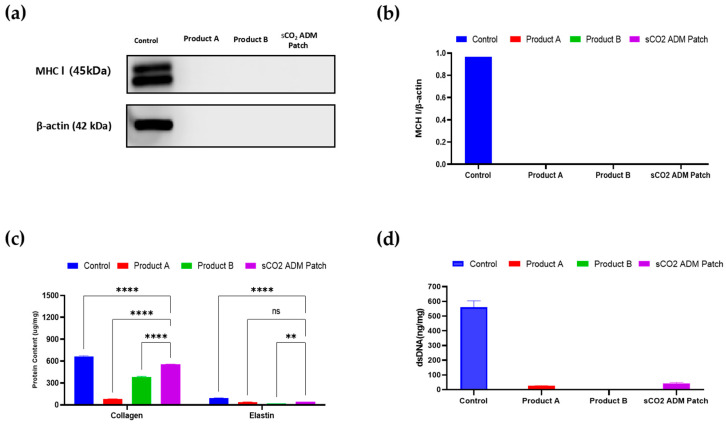
(**a**,**b**) Western blot results of major histocompatibility complex class I (MHC-I). (**c**,**d**) Content of collagen, elastin, and dsDNA in different patches. Data are presented as mean ± SEM. Statistical significance is indicated as follows: ** *p* < 0.01, **** *p* < 0.001, and ns = not significant.

The morphology of the sCO_2_ ADM patch was revealed by SEM images, showing a porous structure at magnifications of 400× and 1000×. And it demonstrated remarkable mechanical properties, exhibiting an ultimate tensile strength of 50.4918 N while those of Product A and Product B were 1.5745 N and 1.3739 N, respectively. The high tensile strength suggested robust structural integrity and resistance of the ADM patch to mechanical deformation during potential clinical applications (Figure 1). In addition, the immunogenic component of patch was removed while collagen and elastin were retained (Figure 2).

### 2.2. Macroscopic and Microscopic Observation of the Wound Healing Process

**Figure 3:**
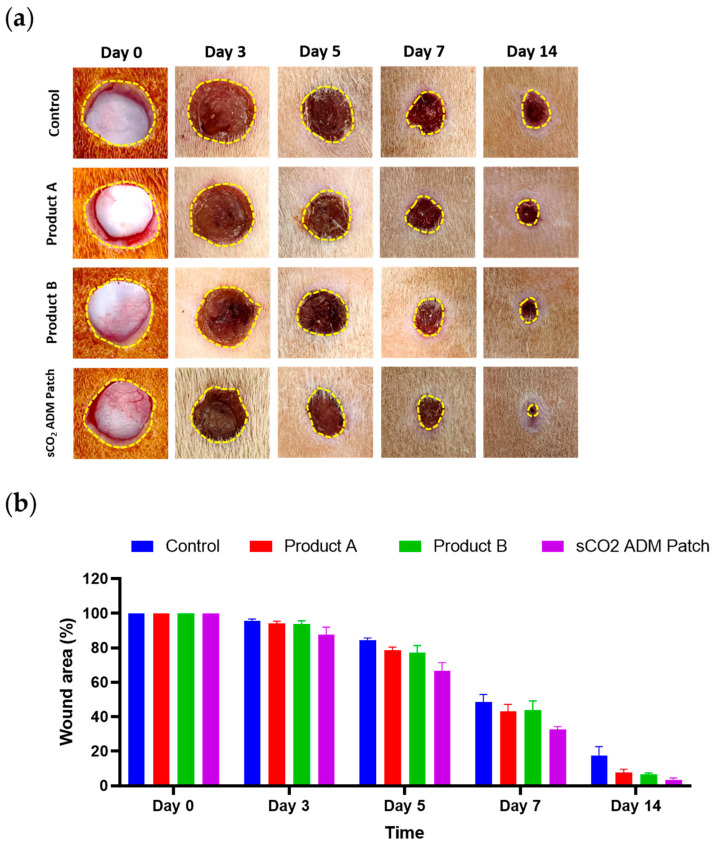
The process of wound healing. (**a**) Representative images of wound healing comparing sCO_2_ ADM patch-treated group and other groups. (**b**) Quantitative measurement of wound recovery over time.

The macroscopic images revealed the recovery of the skin wounds at multiple time points: day 0 and subsequent intervals of 3, 5, 7, and 14 days following wound creation. Throughout the wound healing process, both the treatment groups and the control group showed a reduction in wound area. It was notable that the rats in the three treatment groups exhibited faster wound closure than those in the control group. In particular, from the fifth day onwards, three treatment groups exhibited a significantly smaller wound area compared to the control group. In addition, the rats in the sCO_2_ ADM patch treated group had better closure ratios than those in the other sCO_2_ ADM patch treated groups. The sCO_2_ ADM patch-treated group consistently presented the smallest wound dimensions among all groups (Figure 3).

**Figure 4:**
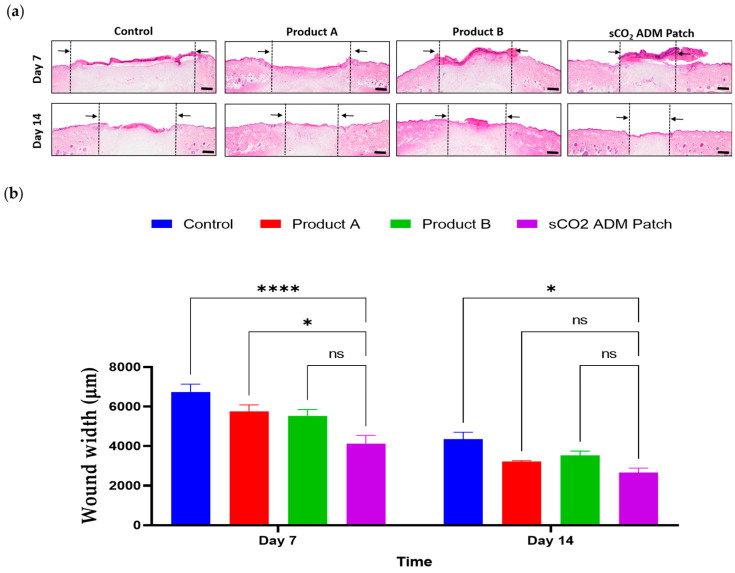
Wound width evaluation over time. (**a**) H&E staining of skin cross-sections from the injury site on day 7 and day 14; scale bar: 1000 µm. (**b**) Quantitative results for wound width on day 7 and day 14. Data are presented as mean ± SEM. Statistical significance is indicated as follows: * *p* < 0.05, **** *p* < 0.001, and ns = not significant.

**Figure 5:**
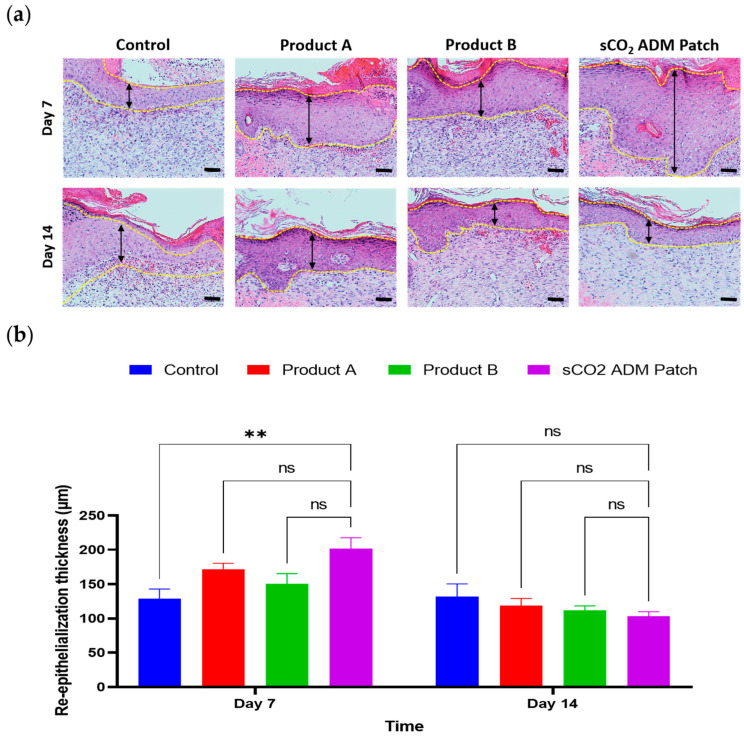
Re-epithelialization status over time. (**a**) H&E staining of epidermal layer cross-sections from the injury site on day 7 and day 14; scale bar: 50 μm. (**b**) Quantitative results for re-epithelialization on day 7 and day 14. Data are presented as mean ± SEM. Statistical significance is indicated as follows: ** *p* < 0.01, and ns = not significant.

Furthermore, hematoxylin and eosin (H&E) staining was used to evaluate the wound width and re-epithelialization. According to the statistical analysis, on day 7, the treatment groups had a significantly shortened wound width compared to the control group. Particularly in the sCO_2_ ADM patch treated group, the average wound width is approximately two-thirds of that in the control group. A similar trend occurred on day 14, where the wound width in the sCO_2_ ADM patch-treated group was remarkably shorter than those in the other groups, and the wounds were almost completely re-epithelialized (Figure 4 and Figure 5).

**Figure 6:**
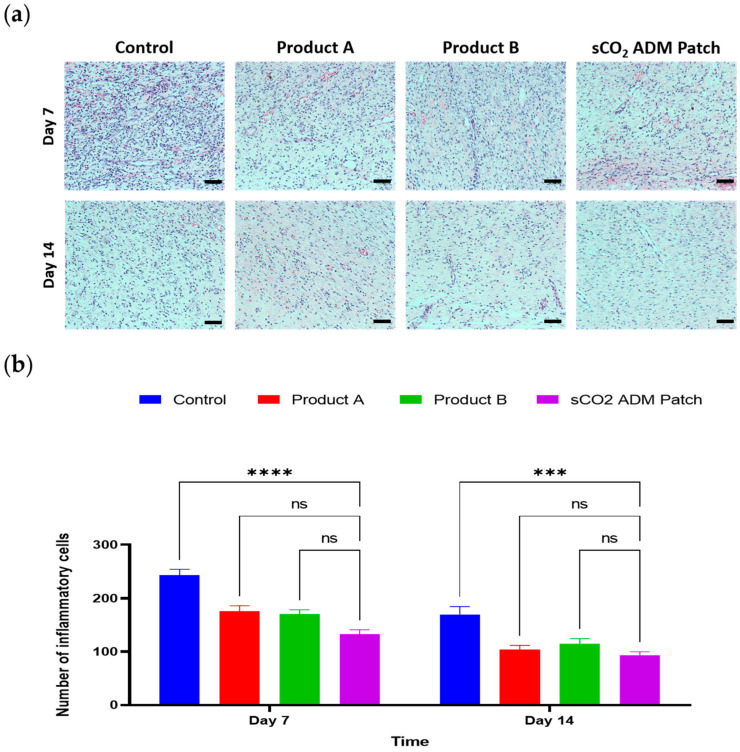
Inflammatory cell counting. (**a**) H&E staining of granulation tissue on from the injury site on day 7 and day 14; scale bar: 50 µm. (**b**) Quantitative results for inflammatory cells on day 7 and day 14. Data are presented as mean ± SEM. Statistical significance is indicated as follows: *** *p* < 0.005, **** *p* < 0.001, and ns = not significant.

**Figure 7:**
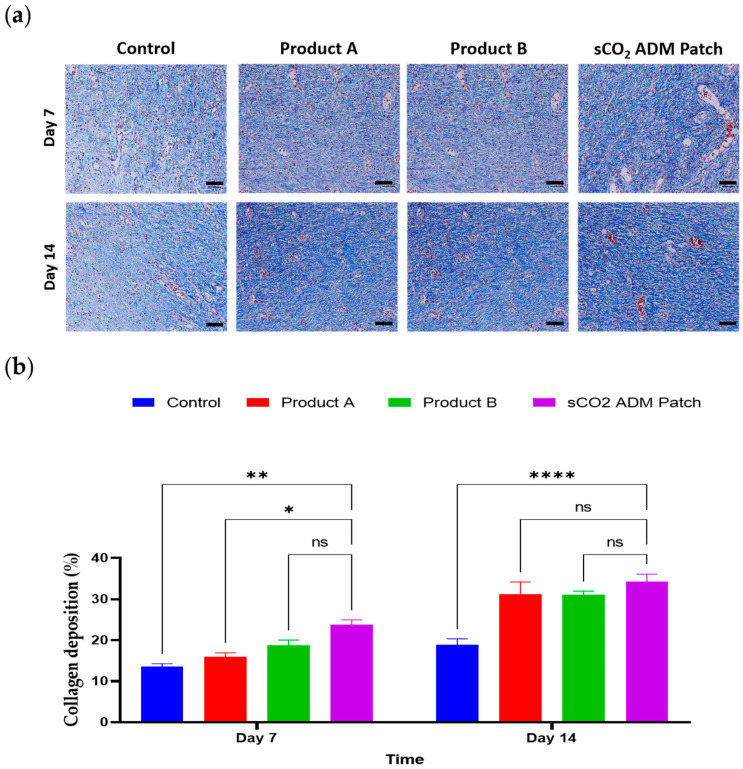
Collagen density analysis. (**a**) MT staining of granulation tissue on from the injury site on day 7 and day 14; scale bar: 50 µm. (**b**) Quantitative results for collagen density on day 7 and day 14. Data are presented as mean ± SEM. Statistical significance is indicated as follows: * *p* < 0.05, ** *p* < 0.01, **** *p* < 0.001, and ns = not significant.

Additionally, as shown in the enlarged HE-stained images, the control group wounds displayed the least granulation tissue proliferation, but with considerable inflammatory cell infiltration. In contrast, the wound areas in the treatment groups showed reduced inflammatory cell infiltration and increased fibroblast proliferation, indicating the progression of wound healing to the proliferative phase. In particular, the sCO_2_ ADM patch-treated group had the lowest inflammatory cell count at both the 7-day and 14-day time points (Figure 6). Moreover, collagen deposition was quantitatively determined using Masson’s trichrome (MT) staining. The wounds treated with the sCO_2_ ADM patch also showed significantly higher collagen deposition than the other treatments (Figure 7).

### 2.3. Effects of the Antiinflammation and Antioxidation by sCO_2_ ADM Patch During the Wound Healing Process

**Figure 8:**
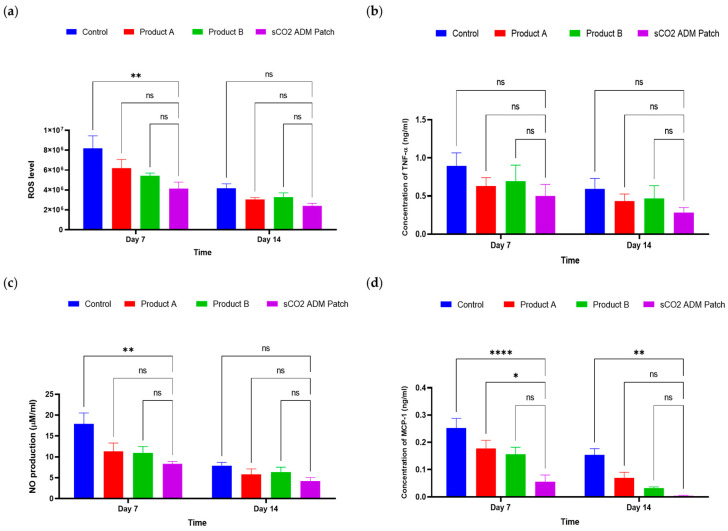
Effects of different treatments on inflammatory and oxidative stress markers on days 7 and 14: (**a**) TNF-α concentration, (**b**) MCP-1 concentration, (**c**) NO production, and (**d**) ROS levels. Data are presented as mean ± SEM. Statistical significance is indicated as follows: * *p* < 0.05, ** *p* < 0.01, **** *p* < 0.001, and ns = not significant.

ELISAs were conducted to assess and quantify the expression of pro-inflammatory cytokines, to evaluate the anti-inflammatory efficacy of sCO_2_ ADM patches. Nitric oxide (NO) and reactive oxygen species (ROS) levels were investigated in wound tissues following treatment with or without ADMs. In Figure 8a, NO production is evaluated, showing a significant reduction (*p* < 0.01) in the sCO_2_ ADM patch groups compared to the control on day 7, but no significant difference is observed by day 14. Figure 8b presents ROS levels, with the sCO_2_ ADM patch group displaying significantly lower levels (*p* < 0.01) on day 7 compared to the control group, while no significant difference is noted on day 14. And in Figure 8c, the concentration of tumor necrosis factor alpha (TNF-α) is shown on days 7 and 14 across the four groups: control, Product A, Product B, and sCO_2_ ADM patch. While the sCO_2_ ADM patch group shows the lowest TNF-α levels at 0.499 ± 0.114 ng/mL and 0.284 ± 0.047 ng/mL on day 7 and day 14, respectively, there is no significant difference (ns) among the groups at both time points. Lastly, Figure 8d depicts monocyte chemoattractant protein 1 (MCP-1) concentrations, where the sCO_2_ ADM patch group exhibits significantly lower levels (0.055 ± 0.018 ng/mL) on day 7 compared to the control groups (*p* < 0.001), with differences persisting at day 14 (*p* < 0.01). Overall, the experimental data suggest that the sCO_2_ ADM patches effectively reduce inflammation and oxidative stress markers.

### 2.4. Acceleration of the Proliferation and Remodeling by sCO_2_ ADM During the Wound Healing Process

**Figure 9:**
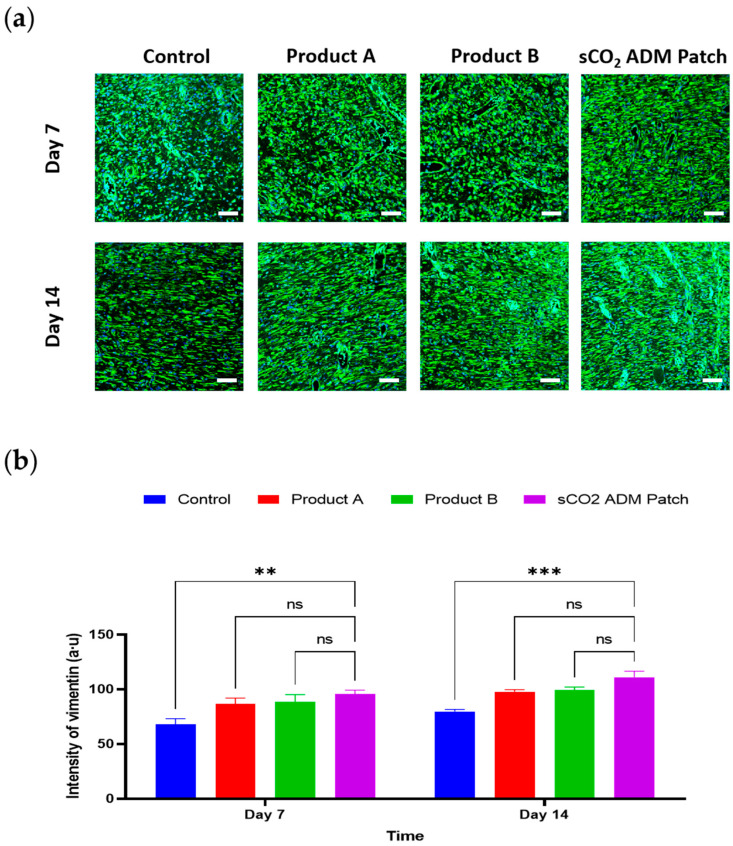
Observations of inflammatory markers in rat wounds using confocal microscopy. Representative immunofluorescence images and quantitative analysis demonstrate: (**a**,**b**) vimentin expression and quantification; scale bar: 50 µm. Data are presented as mean ± SEM. Statistical significance is indicated as follows: ** *p* < 0.01, *** *p* < 0.005, and ns = not significant.

**Figure 10:**
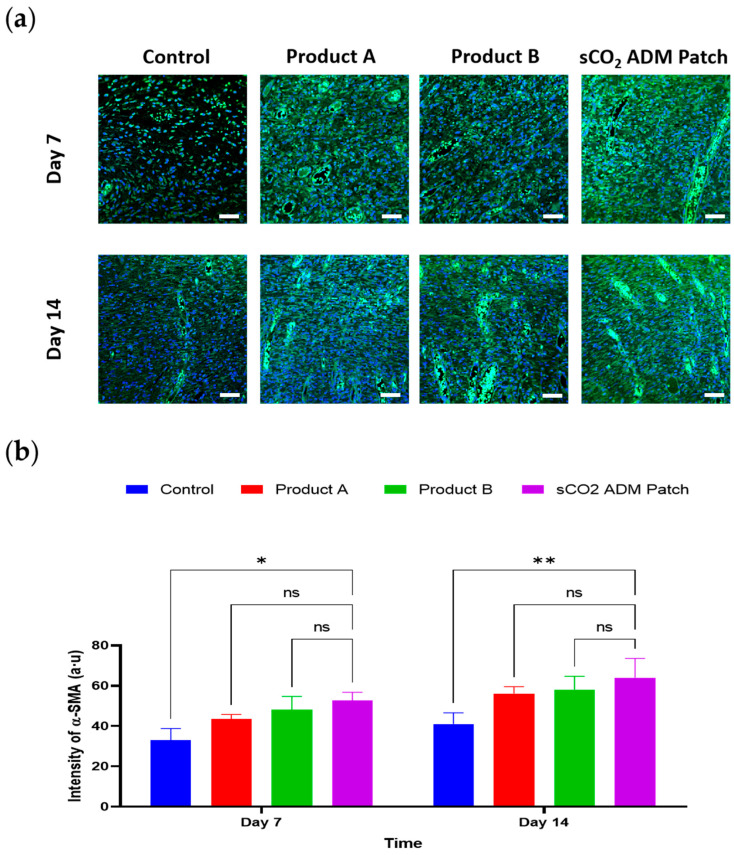
Observations of inflammatory markers in rat wounds using confocal microscopy. Representative immunofluorescence images and quantitative analysis demonstrate: (**a**,**b**) α-SMA expression and quantification; scale bar: 50 µm. Data are presented as mean ± SEM. Statistical significance is indicated as follows: * *p* < 0.05, ** *p* < 0.01, and ns = not significant.

**Figure 11:**
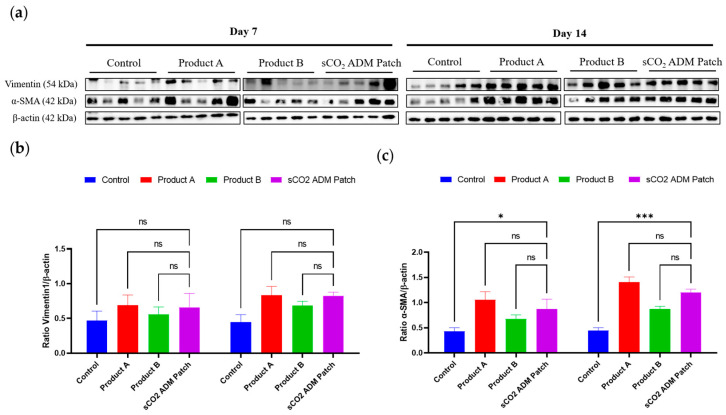
(**a**) Western blot depicting changes in protein levels of vimentin and α-SMA among all groups on Day 7 and Day 14. (**b**,**c**) Densitometric values were normalized against their corresponding protein levels and are presented as mean ± SEM. Statistical significance is indicated as follows: * *p* < 0.05, *** *p* < 0.005, and ns = not significant.

In order to examine the effects of sCO_2_ ADM patch on accelerating proliferation and remodeling, IF staining was conducted to detect various proliferation markers. These IF images correspond to the vimentin and α-SMA, respectively. The analysis of the staining results illustrated that the fluorescence intensity of these proliferation markers was significantly higher in the sCO_2_ ADM patch-treated group compared to other treatment groups and the control group. As shown in Figure 9 and Figure 10, on day 7 the sCO_2_ ADM patch group exhibited the highest intensity at 95.55 ± 2.55. The control group had the lowest intensity, while Product A and Product B displayed moderate levels without significant differences between them. On day 14, vimentin intensity increases further in all groups, with the sCO_2_ ADM patch group maintaining the highest level (111.09 ± 4.16), significantly exceeding all other groups. Similarly, on day 7, the intensity of α-SMA expression was 52.74 ± 1.34 in the sCO_2_ ADM patch-treated group, approximately 15% higher than the other two experimental groups, and over 60% higher than the control group, with these differences being statistically significant. On day 14, α-SMA intensity increases across all groups, with the sCO_2_ ADM patch group showing the highest level. Product B also demonstrates a noticeable increase in intensity compared to the control group, but the enhancement is less pronounced than in the sCO_2_ ADM patch group. Additionally, to establish a more comprehensive assessment of the proliferative capacity of various ADM patches, protein expression analysis was conducted via Western blot. As demonstrated in Figure 11, the expression profiles of vimentin and α-SMA were upregulated in all of the patch treatment groups relative to the control group at both day 7 and day 14 time points.

**Figure 12:**
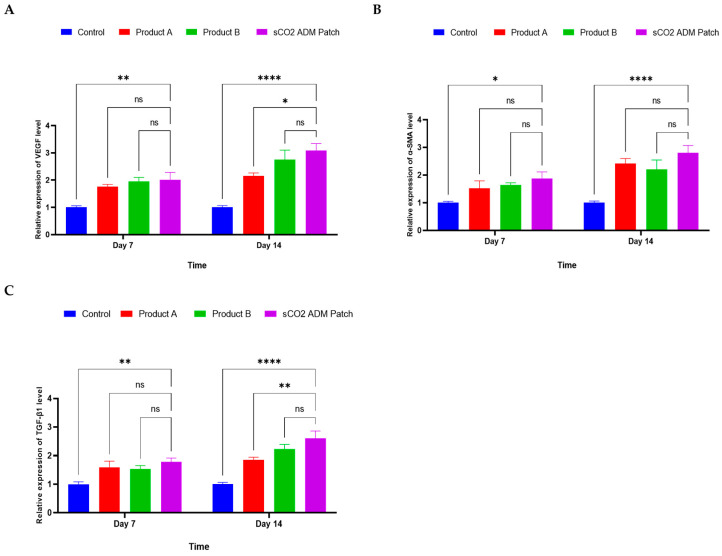
Relative expression levels of different cytokines, (**A**): VEGF, (**B**) α-SMA, (**C**) TGF-β1. Data are presented as mean ± SEM. Statistical significance is indicated as follows: * *p* < 0.05, ** *p* < 0.01, **** *p* < 0.001, and ns = not significant.

RT-PCR was performed to further elucidate and confirm the efficacy of the sCO_2_ ADM patches. The results revealed differential mRNA levels among all groups. Specifically, the sCO_2_ ADM patch-treated group demonstrated elevated mRNA levels of vascular endothelial growth factor (VEGF), α-SMA and transforming growth factor beta 1 (TGF-β1) in the sCO_2_ ADM patch-treated group compared to the other groups on both the 7th and 14th days (Figure 12).

## 3. Discussion

This study demonstrated the efficacy and advantages of the sCO_2_ ADM patch in promoting wound healing, as validated through in vitro, in vivo, and histological evaluations. The findings emphasize the superiority of the sCO_2_ ADM patch.

In ADM patch preparation, tissue decellularization is a critical process designed to preserve the extracellular matrix (ECM)’s structural properties while removing cellular components and immunogenic factors [36,40,69,70,71,72]. This approach aims to minimize potential adverse immune responses and inflammatory reactions after the application of an ADM patch. Researchers have developed multiple decellularization methodologies, including physical techniques such as freeze–thaw cycles, immersion, mechanical agitation, and direct pressure application; chemical approaches involving ionic, nonionic, alcoholic, alkaline, and acidic treatments, as well as chelating and zwitterionic detergents; and biological strategies utilizing antibiotics or enzymatic agents [36,40,70,71,73,74]. Among all the decellularization methodologies, the primary strength of sCO_2_ lies in its exceptional decellularization capabilities. Unlike conventional chemical or enzymatic methods that often compromise ECM structural integrity, sCO_2_ demonstrates remarkable precision in cellular material removal while preserving crucial ECM components [60,75,76,77]. Critically, the method maintains essential proteins such as collagen, glycosaminoglycans, and growth factors, which are fundamental to cellular function and tissue regeneration. The fabrication of powdered sCO_2_ ADM into a patch revealed exceptional structural characteristics. The tensile strength analysis demonstrated an impressive ultimate tensile strength of 56.102 MPa in a dry state and 0.944 MPa in a hydrated state, indicating remarkable mechanical stability. These findings suggest the patch’s remarkable capability to maintain structural integrity during handling, storage, and application, with demonstrated durability across both dry and moist wound environments. The uniform microparticle distribution ensures consistent wound bed interaction, promoting homogeneous tissue repair.

The scanning electron microscope (SEM) analysis revealed a complex microstructural architecture, characterized by porous regions at lower magnifications and dense zones at higher magnifications. This intricate structure offers significant advantages for wound healing, facilitating the controlled exchange of gases and fluids while providing an optimal scaffold that supports cell attachment, migration, and proliferation [78,79]. The patch’s porosity is particularly beneficial for promoting granulation tissue formation and accelerating re-epithelialization. The synergistic combination of sophisticated porosity and robust biocompatibility positions the sCO_2_ ADM patch as a promising solution for both chronic and acute wound management strategies. While this study did not directly examine drug loading and release capabilities, the observed porous structure indicates that the sCO_2_ ADM patch could function as an effective delivery platform for therapeutic agents. Its potential to serve as a reservoir for stem cells, exosomes, or other bioactive molecules—including growth factors, anti-inflammatory cytokines, or antimicrobial peptides—could significantly enhance wound regeneration outcomes, particularly in challenging clinical scenarios such as diabetic ulcers or burn injuries [80,81].

Pro-inflammatory cytokines demonstrate a complex role in wound healing, simultaneously supporting tissue regeneration while potentially compromising healing processes [19,82,83,84,85]. These molecular mediators stimulate keratinocyte proliferation and antimicrobial peptide synthesis, which facilitate acute wound repair; however, sustained or excessive cytokine production can disrupt the healing cascade by prolonging inflammatory responses and impeding tissue restoration [18,23,85,86,87]. Our results revealed that the sCO_2_ ADM patch-treated group exhibited substantially lower levels of pro-inflammatory cytokines compared to the other groups. Similarly, the reduction in ROS and NO levels highlights the antioxidative capacity of the sCO_2_ ADM patch. Oxidative stress is a well-established impediment to wound healing, causing cellular and tissue damage. By effectively mitigating oxidative stress, the patch creates an optimal microenvironment that supports critical wound repair processes, including fibroblast proliferation, collagen synthesis, and angiogenesis. The dual anti-inflammatory and antioxidant effects of the patch make it especially beneficial for treating various wounds.

Wound healing outcomes are critically dependent on fibroblast proliferation and transformation. During tissue repair, activated fibroblasts undergo a pivotal phenotypic transition to myofibroblasts, characterized by the expression of α-SMA. And vimentin is another critical cytoskeletal protein that plays a fundamental role in wound healing by facilitating cell migration and wound closure. Our findings revealed that the sCO_2_ ADM patch-treated group exhibited the highest α-SMA and vimentin expression among all groups. Our findings revealed that the sCO_2_ ADM patch-treated group exhibited the highest α-SMA and vimentin expression among all groups. These findings suggest that the sCO_2_ ADM patch not only promotes faster wound closure but also improves the quality of tissue regeneration, reducing the risk of scar formation. The sCO_2_ ADM patch demonstrates remarkable potential for personalized medicine through its inherent customizability. By modulating parameters such as patch dimensions, thickness, and biochemical properties, the patch can be precisely tailored to address individual patient wound characteristics and healing requirements. The patch’s versatility extends beyond its base structure, offering opportunities for strategic enhancement through targeted biomolecule incorporation. The patch could promote angiogenesis and accelerate wound healing in ischemic wounds by integrating cytokines like vascular endothelial growth factor (VEGF) or platelet-derived growth factor (PDGF).

While the preclinical findings in this study demonstrate significant potential, rigorous clinical validation remains essential to establish the safety and efficacy of the sCO_2_ ADM patch in human subjects. Future research must comprehensively explore the patch’s performance across diverse patient populations and wound types, systematically evaluating critical parameters including healing duration, patient comfort, wound closure rates, and long-term clinical outcomes. To advance the translational potential of this innovative wound healing technology, subsequent studies should focus on multicenter randomized clinical trials, comparative assessments across different wound etiologies, comprehensive safety monitoring, and detailed analyses of functional and esthetic wound healing results. The transition from promising preclinical data to validated clinical intervention requires meticulous, methodical investigation to fully realize the patch’s therapeutic potential.

## 4. Materials and Methods

### 4.1. Supercritical Carbon Dioxide Acellular Dermal Matrix Patch (sCO_2_ ADM Patch) Preparation

Human skin tissue was procured from Seoul Asan Hospital’s tissue donor program, following institutional review board approval (IRB No. 20201305). The specimen underwent preliminary preparation by removing residual adipose tissue and thoroughly rinsing in sterile phosphate-buffered saline (PBS, Thermo Fisher Scientific, Waltham, MA, USA) for approximately 30 min. To isolate the dermis, the tissue was immersed in a 1M sodium chloride solution (prepared in sterile distilled water) at 37 °C for overnight incubation, after which the epidermis was carefully detached from the dermal layer using surgical forceps.

A custom-designed supercritical carbon dioxide (sCO_2_) extraction apparatus, featuring a 0.3 L cylindrical vessel, was utilized for tissue processing. Prior to extraction, the dermis was soaked in PBS supplemented with a 1% antibiotic-antimycotic solution and maintained on an orbital rocker at 30 rpm overnight. The carbon dioxide flow was precisely regulated using a gas flowmeter, with ethanol at 50% concentration introduced as a cosolvent. The extraction was conducted under controlled pressure conditions at 300 bar, with a total processing duration of approximately 3 h.

Following the sCO_2_ extraction, the acellular dermal matrix (ADM) was stored at −80 °C for 12 h and then freeze-dried for 48 h to remove moisture from the dermal matrix, facilitating particle processing. The freeze-dried sCO_2_ ADM was converted into spherical powder-like microparticles with diameters ranging from 150 to 300 microns using an ultrasonic pulverization mechanism. The microparticles were combined with sterilized distilled water at a 2% mass ratio and homogenized using a magnetic stirrer at 100 RPM for 10 min to create a preliminary mixture. This mixture was then introduced into a high-pressure homogenizer and subjected to three sequential high-pressure dispersion cycles utilizing a 200 μm nozzle chamber at 15,000 psi. The final dispersion was carefully transferred to a 100 cm^2^ dish to ensure a consistent coating. The dish was subsequently positioned in a vacuum oven, maintained at 40 °C under a vacuum of −0.9 bar, and dried for a whole day. Upon conclusion of the drying process, a refined ADM patch was successfully obtained.

### 4.2. Scanning Electron Microscope (SEM) Imaging for sCO_2_ ADM Patch Morphology

The surface morphology of the sCO_2_ ADM patch was analyzed using a scanning electron microscope (SEM, IM-150, Aro Optics, Ulsan, Korea). During the surface analysis, the sample was coated with a thin layer of gold and photographed at 5 kV to observe the surface features.

### 4.3. Tensile Strength Testing

The tensile strength of sCO_2_ ADM patch was measured at a dimension of 1 cm × 5 cm at a strain rate of 10mm and 4mm, respectively, using a universal testing machine (AGS-X, Shimadzu, Kyoto, Japan). The tensile strength was evaluated in both hydrated and dry states.

### 4.4. Immunogenicity Testing and Component Determination

Major histocompatibility complex class I (MHC I) expression was assessed via Western blot. Protein samples extracted from the acellular dermal matrix (ADM) powder solution were separated by SDS-PAGE and transferred to nitrocellulose membranes. The membranes were blocked with 5% skimmed milk, then incubated with primary antibody overnight at 4 °C, followed by secondary antibody application for 2 h. Protein bands were visualized using the ChemiDoc^TM^ Imaging System (Bio-Rad Laboratories, Hercules, CA, USA). Target protein expression levels were semi-quantified and normalized to beta-actin using ImageJ version 1.52 (NIH, Bethesda, MD, USA). The content of collagen and elastin were evaluated by Biocolor Sircol Kit (Biocolor Ltd., Belfast, UK) after freezing all samples. DNA was extracted using the Dneasy blood & tissue kit (Qiagen, Hilden, Germany) and dsDNA was quantified by Qubit dsDNA HS assay kit (Thermo Fisher Scientific, Waltham, MA, USA).

### 4.5. Animal Experiment

The experimental protocol involving animal subjects was meticulously designed and executed in full compliance with the ethical standards established by the Institutional Animal Care and Use Committee of Seoul National University Bundang Hospital (approval number: BA-2404-389-010). The research cohort consisted of eight-week-old male Sprague–Dawley rats, procured from BioOrient Company (Seongnam, Republic of Korea), with a body weight range of 250–350 g. Throughout the study, animals were housed in pairs within a controlled, specific-pathogen-free (SPF) environment, provided with ad libitum access to standard laboratory nutrition and water. The vivarium conditions were precisely regulated, maintaining a consistent 12 h light/dark photoperiod, ambient temperature of 24 °C, and relative humidity of 55%, thereby establishing optimal physiological parameters for the experimental subjects.

Sprague–Dawley adult male rats were randomly divided into four groups of 10 rats, with three groups being applied different ADM patches and one control group. Product A and B are commercial ADM products generated by chemical decellularization processes. Full-thickness excisional wounds measuring 10 mm in diameter were created using a modified biopsy punch on the dorsal skin of rats after anesthesia with Isoflurane. Acellular dermal matrices of uniform size (0.785 cm^2^) were applied to wounds with each wound sealed using Tegaderm film (3M, Maplewood, MN, USA) and then fixed with medical tape. Macroscopic observations of wounds were conducted and documented photographically using a digital camera on days 0, 3, 5, 7, and 14. On the designated experimental timepoints of days 7 and 14, half of the rats from each experimental cohort were humanely euthanized through controlled CO_2_ inhalation, a standard method for minimizing animal distress during terminal procedures. Following euthanasia, dermal tissue specimens were collected, comprising the entire full-thickness circular wound area, which were precisely excised using sterile 12 mm diameter punch biopsy tools to ensure consistent and uniform sample acquisition.

### 4.6. Measurement of the Wound Healing Area

The wound closure percentage was calculated using the equation:

Wound closing area (%) = [(A0 − Ai)/A0] × 100%

where A0 represents the wound area on day 0, and Ai represents the wound area on subsequent days (0, 3, 5, 7, 10, and 14). Wound regions were measured using the ImageJ version 1.52 (NIH, Bethesda, MD, USA).

### 4.7. Histological Analysis

Tissue samples were fixed in 10% neutral buffered formalin, progressively dehydrated through increasing ethanol concentrations (80% to 100%), embedded in paraffin, and sectioned into 5 µm thick slices. A histological assessment of rat skin wounds was conducted to evaluate the quality of wound healing at the tissue level. Through detailed histological analysis, three key aspects of wound repair were examined: granulation tissue formation, epidermis layer reconstruction, and collagen deposition and inflammation.

### 4.8. Hematoxylin and Eosin (H&E) Staining

The tissue sections were deparaffinized in xylene for 3 min and then rehydrated using a graded ethanol series. This rehydration protocol involved two 3 min immersions in 100% ethanol, followed by sequential 3 min treatments in 95%, 90%, 80%, and 70% ethanol solutions. The sections were then washed twice with distilled water for 3 min each. The staining procedure began with hematoxylin for 5 min, followed by two 3 min distilled water rinses to remove excess stain. The sections were then briefly incubated in Bluing reagent for 10–15 s. Subsequently, the tissue slices were rinsed with distilled water and stained with Eosin for 30 s. Dehydration was completed through three successive 3 min immersions in 95% and 100% ethanol, respectively. Finally, the tissue sections were cleared through 3 changes in Xylene for 1 min each and coverslipped. H&E-stained slides were examined using an Olympus optical microscope.

### 4.9. Masson’s Trichrome (MT) Staining

The tissue slides were stained according to the manufacturer’s instructions using Masson’s trichrome staining kit. The procedure involved incubation with Bouin solution at room temperature overnight, followed by thorough rinsing with distilled water until the tissue slides became completely transparent. The staining protocol proceeded as follows: first, tissue slices were stained with Weigert’s hematoxylin for 10 min, and then rinsed with distilled water. Next, the sections were immersed in Biebrich scarlet acid solution for 10 min. After rinsing with distilled water, the tissue samples underwent a 10 min staining process in phosphomolybdic–phosphotungstic acid solution, followed by another 10 min staining with aniline blue solution. The specimens were then washed with distilled water and immersed in acetic acid for 3 min. Dehydration was performed using a graded ethanol series: 3 min in 100% ethanol, 2 min in 95% ethanol, and finally 10 min in Xylene. Each tissue slide was mounted with a single droplet of mounting media. The synthesis and intensity of collagen fibers were visualized using a microscope and quantified using ImageJ version 1.52 (NIH, Bethesda, MD, USA).

### 4.10. Immunofluorescence (IF) Staining

Tissue sections were deparaffinized, hydrated, and blocked with 4% BSA in PBS at room temperature for one hour to prevent non-specific binding. The tissue slides were then incubated overnight at 4 °C with primary antibodies against vimentin, and α-SMA, (Santa Cruz Biotechnology, Dallas, TX, USA). After washing, the sections were treated with Alexa Fluor 488 goat anti-mouse secondary antibody (Invitrogen, Thermo Fisher Scientific, MA, USA) for one hour at room temperature in the dark. Nuclear labeling was performed using DAPI (Fluoroshield with DAPI, Sigma-Aldrich, St. Louis, MO, USA). Fluorescent signals were captured with a Zeiss LSM 710 confocal microscope (ZEISS group, Oberkochen, Germany) and analyzed using ImageJ version 1.52 (NIH, Bethesda, MD, USA) to quantify biomarker intensity, including inflammatory cytokines and proliferation markers, enabling a comparison of expression levels.

### 4.11. Protein Preparation

On days 7 and 14, the tissues were ground and immersed in a protein solution containing a protease inhibitor cocktail and 1 mM phenylmethylsulfonyl fluoride (PMSF) for 60 min on ice and vortexed for an interval of 15 min each time. Tissue homogenates underwent high-speed centrifugation at 14,000 rpm for 15 min, and were maintained at 40 °C to separate cellular debris from the protein-rich supernatant. The isolated protein solution was immediately aliquoted and stored at −80 °C, ensuring protein stability for subsequent analytical techniques, including the enzyme-linked immunosorbent assay (ELISA) and the nitric oxide quantification and 2′,7′-dichlorofluorescein diacetate (DCF-DA) assays.

### 4.12. Western Blot

Vimentin and alpha-smooth muscle actin (α-SMA) expression in the wound tissues were evaluated by Western blot on days 7 and 14. Protein samples were separated by SDS-PAGE and transferred to nitrocellulose membranes. After blocking with 5% skimmed milk, membranes were incubated overnight at 4 °C with primary antibodies (Santa Cruz Biotechnology, Dallas, TX, USA), followed by a 2 h incubation with a secondary antibody (H+L)-HRP conjugate (Bio-Rad Laboratories, Berkeley, CA, USA). Protein bands were visualized using the ChemiDo^TM^ Imaging System (Bio-Rad Laboratories, Berkeley, CA, USA). Expression levels of target proteins were semi-quantified and normalized to beta-actin using ImageJ version 1.52 (NIH, Bethesda, MD, USA).

### 4.13. Enzyme-Linked Immunosorbent Assay (ELISA)

For the ELISA analysis, 96-well plates were coated overnight at 4 °C with capture antibodies against tumor necrosis factor alpha (TNF-α) and monocyte chemoattractant protein 1 (MCP-1) from BD Biosciences (Franklin Lakes, NJ, USA), diluted 1:500 in PBS. After coating, the plate was washed and blocked with a blocking buffer containing 10% FBS in PBS to prevent non-specific binding. Standards and supernatant samples were diluted with the blocking buffer, added to the wells, and incubated for 3 h at 25 °C. A second antibody (1:500 in blocking buffer, BD Biosciences, Franklin Lakes, NJ, USA) was added for a further 2 h incubation. Following another wash, avidin–peroxidase (Sigma-Aldrich, MO, USA) was applied for 30 min. Subsequently, 100 µL of a 1:1 solution of substrate reagents A and B (BD OptEIA TMB Substrate Reagent Set, BD Biosciences, Franklin Lakes, NJ, USA) was added to each well. The absorbance was measured at 405 nm using a BioTek Epoch 2 microplate spectrophotometer (BioTek Instruments, Winooski, VT, USA) for quantitative analysis of the specific proteins. The standard curve was prepared using 12 points with a 2-fold dilution starting from 10 ng/mL of recombinant Rat TNF-α and MCP-1 proteins from BD Biosciences (Franklin Lakes, NJ, USA).

### 4.14. Nitric Oxide Quantification

Nitric oxide (NO) oxidation products in the cell supernatant were analyzed using the modified Griess reagent (Sigma-Aldrich, St. Louis, MO, USA). A total of 50 µL of the supernatant was mixed with 50 µL of 1× Griess reagent (modified) and incubated at room temperature for 15 min. The reagent was prepared by dissolving 10 g of Griess reagent (modified) in 250 mL of water and inverting the bottle for five minutes, as per the manufacturer’s instructions. Nitrite standard solutions, prepared via 2-fold dilutions ranging from 0 to 50 µM, were used to generate a standard curve for quantification. Absorbance was measured at 504 nm using a BioTek Epoch 2 microplate spectrophotometer (BioTek Instruments, Winooski, VT, USA).

### 4.15. 2′,7′-Dichlorofluorescein Diacetate (DCF-DA) Assay

The production of reactive oxygen species (ROS) was comprehensively quantified using a DCF-DA kit (Abcam, Cambridge, United Kingdom). Protein extracts, standardized to a concentration of 30 μg/mL across all experimental groups, were systematically combined with a 20 μM DCF-DA fluorescent probe. The reaction mixture was subsequently incubated under controlled conditions—maintained at 37 °C in a 5% CO_2_ atmosphere—and protected from light for a precise 45 min interval. Fluorescence intensity was quantitatively assessed utilizing a multi-microplate spectrophotometric reader (SpectraMax id5, Avantor, Radnor, PA, USA), with excitation and emission wavelengths set at 438/535 nm.

### 4.16. RT-PCR

Total RNA extraction from harvested skin tissue was conducted utilizing RNAiso Plus reagent (Takara Bio, Shiga, Japan), meticulously following the manufacturer’s recommended protocol. Approximately 1 µg of purified total RNA was then subjected to complementary DNA (cDNA) synthesis using the RevertAid First Strand cDNA Synthesis Kit (Thermo Fisher Scientific, MA, USA). The specific primer pairs used to analyze the expression of Collagen I, TGF-β1, VEGF, and GAPDH were as follows: Collagen I forward 5′-CCGTGACCTCAAGATGTGC-3′, Collagen I reverse 5′-GAACCTTCGCTTCCATACTCG-3′, TGF-β1 reverse 5- CCTCGACGTTTGGGACTGAT-3′, TGF-β1 forward 5′-TGACGTCACTGGAGTTGTCC -3′, VEGF reverse 5′-GCTGGCTTTGGTGAGGTTTG-3′, VEGF forward 5′- GCTGCAATGATGAAGCCCTG-3′, GAPDH forward 5′-CGCTAACATCAAATGGGGTG-3′, GAPDH reverse 5′-TTGCTGACAATCTTGAGGGAG-3′. For quantitative PCR, TB Green^®^ Premix Ex Taq™ II (Takara Bio, Shiga, Japan) was used on the QuantStudio™ 3 Realtime PCR (Thermo Fisher Scientific, Waltham, MA, USA) with the following PCR conditions: initial denaturation at 95 °C for 30s, followed by 45 cycles of amplification at 95 °C for 5s and 60 °C for 34s. Relative mRNA expression values were determined using the comparative CT method, the 2^−ΔΔCT^ method.

### 4.17. Statistics Analysis

All experimental data were collected through measurements and presented as the mean ± standard error of the mean (SEM). Statistical analysis was performed using GraphPad Prism version 9 (GraphPad Software Inc., San Diego, CA, USA), with group differences evaluated using a two-way ANOVA test. A *p*-value less than 0.05 was considered statistically significant, with significance levels denoted as follows: * *p* < 0.05, ** *p* < 0.01, *** *p* < 0.005, **** *p* < 0.001.

## 5. Conclusions

This study demonstrates the significant potential of the sCO_2_ ADM patch as an innovative wound healing solution. The patch exhibited superior structural properties, including high tensile strength, uniform thickness, and porous architecture that facilitates cell migration and tissue integration. In vitro analyses confirmed excellent biocompatibility and safety, while in vivo studies revealed accelerated wound closure, reduced inflammation, enhanced collagen deposition, and upregulated markers of cellular proliferation and tissue remodeling. These results indicate that the sCO_2_ ADM patch effectively supports all wound healing phases by creating an optimal microenvironment for skin regeneration. Despite these promising outcomes, additional research is needed to assess long-term performance, safety, and efficacy across diverse wound types, including chronic, diabetic, and burn injuries. Future investigations should prioritize clinical translation through large-scale manufacturing development, quality control protocols, sterilization validation, and regulatory approval processes. Exploring functional enhancements, such as drug loading capabilities or growth factor incorporation, could further expand clinical applications. The sCO_2_ ADM patch represents a promising next-generation biomaterial for advanced wound care, offering strong potential for both scalable production and successful clinical implementation.

## Data Availability

All data generated or analyzed in this study are available from the corresponding author on reasonable request.

## Abbreviations

The following abbreviations are used in this manuscript:

ADM	acellular dermal matrix
α-SMA	alpha smooth muscle actin
DCF-DA	2′,7′-dichlorofluorescein diacetate
dECM	decellularized extracellular matrix
ECM	extracellular matrix
ELISA	enzyme-linked immunosorbent assay
H&E	hematoxylin and eosin
IF	immunofluorescence
MCP-1	monocyte chemoattractant protein 1
MHC	major histocompatibility complex
MT	Masson’s trichrome
NO	nitric oxide
PDGF	platelet-derived growth factor
PMSF	phenylmethylsulfonyl fluoride
ROS	reactive oxygen species
sCO_2_	supercritical carbon dioxide
SEM	scanning electron microscope
SPF	Specific pathogen free
TGF-β1	transforming growth factor-beta 1
TNF-α	tumor necrosis factor alpha
VEGF	vascular endothelial growth factor
